# Addition of* Wollastonite* Fibers to Calcium Phosphate Cement Increases Cell Viability and Stimulates Differentiation of Osteoblast-Like Cells

**DOI:** 10.1155/2017/5260106

**Published:** 2017-08-21

**Authors:** Juliana Almeida Domingues, Mariana Motisuke, Celso Aparecido Bertran, Moema A. Hausen, Eliana Aparecida de Rezende Duek, José Angelo Camilli

**Affiliations:** ^1^Department of Structural and Functional Biology, Biology Institute, University of Campinas (UNICAMP), Campinas, SP, Brazil; ^2^Department of Physiological Science, Biomaterials Laboratory, Pontifical Catholic University of Sao Paulo (PUC-SP), Sorocaba, SP, Brazil; ^3^Bioceramics Laboratory, Science and Technology Institute, Federal University of São Paulo (UNIFESP), São José dos Campos, SP, Brazil; ^4^Department of Physical Chemistry, Chemistry Institute, University of Campinas (UNICAMP), Campinas, SP, Brazil; ^5^Department of Materials Engineering, Faculty of Mechanical Engineering, University of Campinas (UNICAMP), Campinas, SP, Brazil

## Abstract

Calcium phosphate cement (CPC) that is based on *α*-tricalcium phosphate (*α*-TCP) is considered desirable for bone tissue engineering because of its relatively rapid degradation properties. However, such cement is relatively weak, restricting its use to areas of low mechanical stress.* Wollastonite* fibers (WF) have been used to improve the mechanical strength of biomaterials. However, the biological properties of WF remain poorly understood. Here, we tested the response of osteoblast-like cells to being cultured on CPC reinforced with 5% of WF (CPC-WF). We found that both types of cement studied achieved an ion balance for calcium and phosphate after 3 days of immersion in culture medium and this allowed subsequent long-term cell culture. CPC-WF increased cell viability and stimulated cell differentiation, compared to nonreinforced CPC. We hypothesize that late silicon release by CPC-WF induces increased cell proliferation and differentiation. Based on our findings, we propose that CPC-WF is a promising material for bone tissue engineering applications.

## 1. Introduction

Calcium phosphate cement (CPC) possesses excellent biocompatibility and osteoconductivity* in vivo *and is commonly used as a biomaterial in bone implants [1]. CPC is made of calcium phosphates, such as *α*-tricalcium phosphate (*α*-TCP) or *β*-tricalcium phosphate (*β*-TCP), which in contact with water dissolves and precipitates into calcium deficient hydroxyapatite [2, 3]. The resulting degradation products of CPC, such as calcium and phosphorus, induce the bioactivity of the material [3, 4], although the poor mechanical properties restrict its applications to small oral maxillofacial defects and the covering of metallic prostheses [5, 6].

In trials aimed at improving the mechanical properties of CPC, small and elongated fibers and whiskers have been applied as a reinforcing material [7]. The reinforcement would be made of calcium phosphate compounds (hydroxyapatite whiskers and fibers) [8], calcium carbonates (aragonite whiskers) [9], and calcium silicates (CaSiO_3_,* wollastonite* whiskers, and fibers [WF]) [10]. Recent studies suggest that the addition of inorganic silicon compounds to biomaterials such as hydroxyapatite and bioactive glasses might influence the metabolism of osteoblast-like cells involved in the process of mineralization [11, 12]. Also, solutions containing a high concentration of inorganic silicon compounds stimulate the expression of genes related to bone activity, enabling bone neoformation by osteoblast-like cells [13].

The mechanical properties of *α*-TCP-based CPC can be enhanced by the addition of WF (CaSiO_3_) [14]. This raises the possibility of increasing the bioactivity of CPC through the release of silicon during* wollastonite* hydrolysis. Motisuke et al. [14] found that addition of 5% (w/w) WF reinforces the compressive strength of an apatite CPC by 250% compared to nonreinforced CPC (from 14.5 to 50.4 MPa).* Wollastonite* exhibits excellent* in vitro* bioactivity [15], as demonstrated by the relatively rapid formation of an apatite layer on its surface compared to other bioactive materials (e.g., bioactive glass). Formation of this apatite layer is essential for integration of the implanted material to the surrounding bone, favoring the proliferation and activity of osteoblast-like cells [16].

The purpose of the present study was to compare the cytocompatibility of CPC and CPC-WF* in vitro*. The study evaluated the potential of the two types of cement as a substrate for the differentiation, adhesion, and proliferation of osteoblast-like cells.

## 2. Material and Methods

### 2.1. Material

*α*-TCP was obtained by solid-state reaction as published elsewhere [17]. Briefly, a stoichiometric mixture of Mg-free calcium carbonate (CaCO_3_) and Mg-free monetite (CaHPO_4_) was calcined at 1300°C and milled to achieve a granulometric distribution of 1.33 to 10 *μ*m and a mean particle size of 4.93 *μ*m. WF were synthesized by the salt fusion method [14]. In summary, calcium carbonate (CaCO_3_, Sigma Aldrich) and electronic grade silicon dioxide (fumed SiO_2_, Sigma Aldrich) were intimately mixed with a NaCl/KCl flux and calcined at 950°C and salt was washed out with deionized water. To prepare CPC-WF, *α*-TCP and WF were mixed at a *α*-TCP to WF ratio of 5% (w/w). This 5% WF ratio was assumed optimal for increasing the mechanical properties of CPC, as shown in our previous work [14]. CPC composite disks (WF reinforced [CPC-WF] and nonreinforced [CPC]) were autoclaved and immersed in sterile culture medium. All disks were 0.8 mm in diameter. Before cell seeding, all CPC and CPC-WF disks were immersed in Dulbecco's modified Eagle's medium (DMEM) for 3 days, with medium replaced daily. This was to achieve ion balance with the culture medium. The daily medium change was necessary to stabilize the balance of essential ions, as previously reported [18].

### 2.2. Isolation of Osteoblast-Like Cells

Osteoblast-like cells were obtained from the calvaria of 20-day-old Lewis rats by the explant method. This experimental protocol was approved by the Ethics Committee on Animal Use of UNICAMP (CEUA 2606-01). Before each cell isolation procedure, animals were sacrificed, and the calvaria is removed and immersed in DMEM (Sigma Aldrich, catalog number D5796) supplemented with antibiotics (Sigma Aldrich, catalog number A5955). The soft tissue was removed with a scalpel. Next, the calvaria was fragmented and immersed in flasks containing DMEM supplemented with antibiotics and 10% fetal bovine serum (Sigma Aldrich, catalog number 2442). The culture flasks were kept at 37°C in a 5% CO_2_ atmosphere. The medium was changed every 2-3 days and cells were used for the experiments until the fourth passage.

### 2.3. Cell Viability

Cell viability was evaluated after 1, 7, and 14 days in culture. After the ion balance period (described above), cells were seeded on the materials at a concentration of 3 × 10^4^ cells per disk. As negative controls, cells were cultured in a polystyrene 24-well plate. After incubation, viable cells were evaluated using a colorimetric MTT (3-(4,5-dimethylthiazol-2-yl)-2,5-diphenyl tetrazolium bromide) (Sigma Aldrich, catalog number 88417) reduction assay for mitochondrial activity (Sigma Aldrich, USA). After 4 h of incubation in the presence of MTT, absorbance readings were made using a microplate reader (model Elx-800-UV, Bio-Tek Instruments, USA) at a wavelength of 570 nm.

### 2.4. Scanning Electron Microscopy

Cell morphology on CPC disks was analyzed by scanning electron microscopy (JEOL JXA-840A). For this purpose, samples were fixed in a solution of 4% paraformaldehyde (Prolab, catalog number 01P1005.01.A), 2.5% glutaraldehyde (Millipore, catalog number 104239), 0.03% picric acid (VETEC QUÍMICA FINA LTDA, catalog number 000910.06), and 1% tannic acid (Synth, catalog number 01A2012.01.AF) in DMEM for 30 min. Next, samples were washed in Phosphate Buffer Saline (PBS), postfixed in 1% osmium tetroxide (Sigma Aldrich, catalog number, 419494), and dehydrated in an increasing ethanol series. Samples were then critical-point dried (Balzers CTD 030) and sputtered with gold (Balzers SCD 050).

### 2.5. Alkaline Phosphatase

Alkaline phosphatase (ALP) activity was assayed to identify the early stage of osteoblast differentiation in the various materials. ALP activity can indicate whether cells are osteoblast-like cells. The colorimetric method used was based on the conversion of p-nitrophenyl phosphate into p-nitrophenol in the presence of ALP. Cells were seeded on disks with DMEM supplemented with 3 mM *β*-glycerol phosphate, 0.1 mM ascorbic acid, and 1 nM dexamethasone. ALP activity was measured at 7, 10, and 14 days after incubation. Before the assay, disks were washed twice with PBS and cells were lysed by sonication and Triton X-100, according to the manufacturer's instructions (SensoLyte® pNPP Alkaline Phosphatase Assay Kit, Anaspec, Inc.). Production of p-nitrophenol was measured by absorbance in a microplate reader (model Elx-800-UV, Bio-Tek Instruments, USA) at a wavelength of 405 nm.

### 2.6. Inductively Coupled Plasma Optical Emission Spectroscopy (ICP-OES)

To determine Ca^2+^, Si, and P ion levels, aliquots of medium were removed during the ion balance period (during days 1 and 3 of culture) and during cell culture (at days 1, 7, and 14) and analyzed by ICP-OES (Perkin Elmer, Optima 3000DV).

### 2.7. Statistical Analysis

All data were compared by one-way analysis of variance (ANOVA). Whenever statistically significant differences (*p* < 0.05) were identified, Tukey's post hoc test was applied (BioEstat, version 5.0). All experiments were performed in quintuplicate.

## 3. Results

### 3.1. Cell Viability

**Figure 1:**
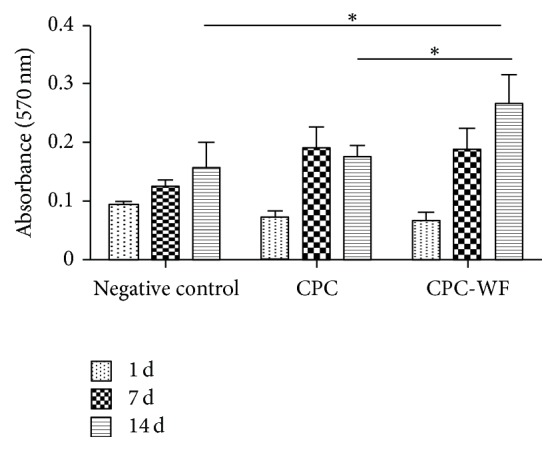
MTT assays after 1, 7, and 14 days of cell culture on CPC disks. Data are expressed as means and standard deviation. *p* values of <0.01 are indicated by an asterisk.

In all samples tested, the cell viability assay showed increased cell metabolic activity over time. However, significantly higher cell activity (*p* < 0.01) was observed on CPC-WF than on either polystyrene plates (negative control) or CPC (Figure 1).

### 3.2. Cell Morphology

**Figure 2:**
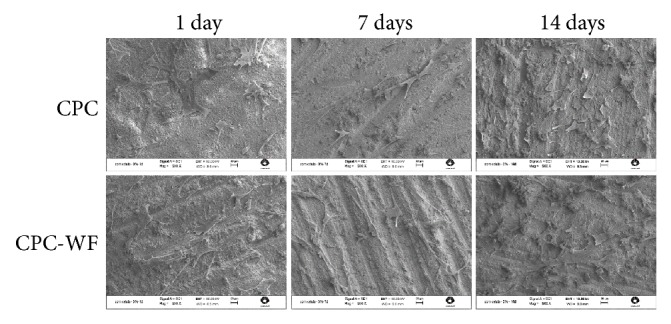
Scanning electron micrographs of CPC and CPC-WF disks after 1, 7, and 14 days of culture. Osteoblast-like cells were well adhered, and the topography of the material did not interfere with cell adhesion.

Scanning electron microscopy showed that cells were able to adhere and spread on the tested samples (Figure 2). Cytoplasmic prolongations were observed in all samples after 1, 7, and 14 days of culture.

### 3.3. Alkaline Phosphatase Activity

**Figure 3:**
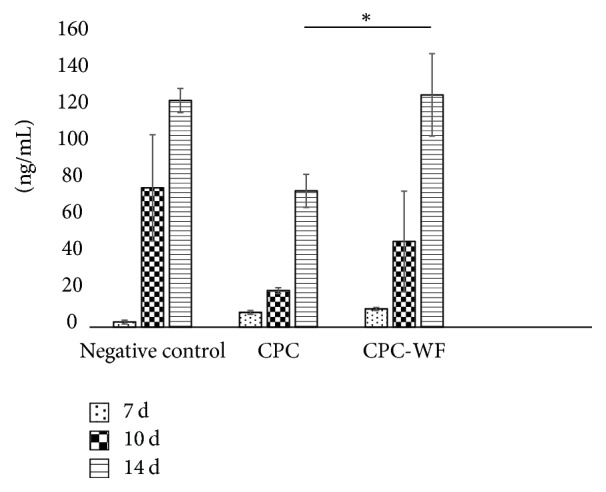
Alkaline phosphatase activity after 7, 10, and 14 days of culture. Negative control represents cells induced to differentiate and cultured in the well plate. *p* values of <0.01 are indicated by an asterisk.

ALP activity increased over time in all samples and was significantly higher in CPC-WF samples than CPC samples after 14 days of cell culture (*p* < 0.05) (Figure 3).

### 3.4. Concentration of Ca^2+^, Si, and P Ions in Culture Medium

**Figure 4:**
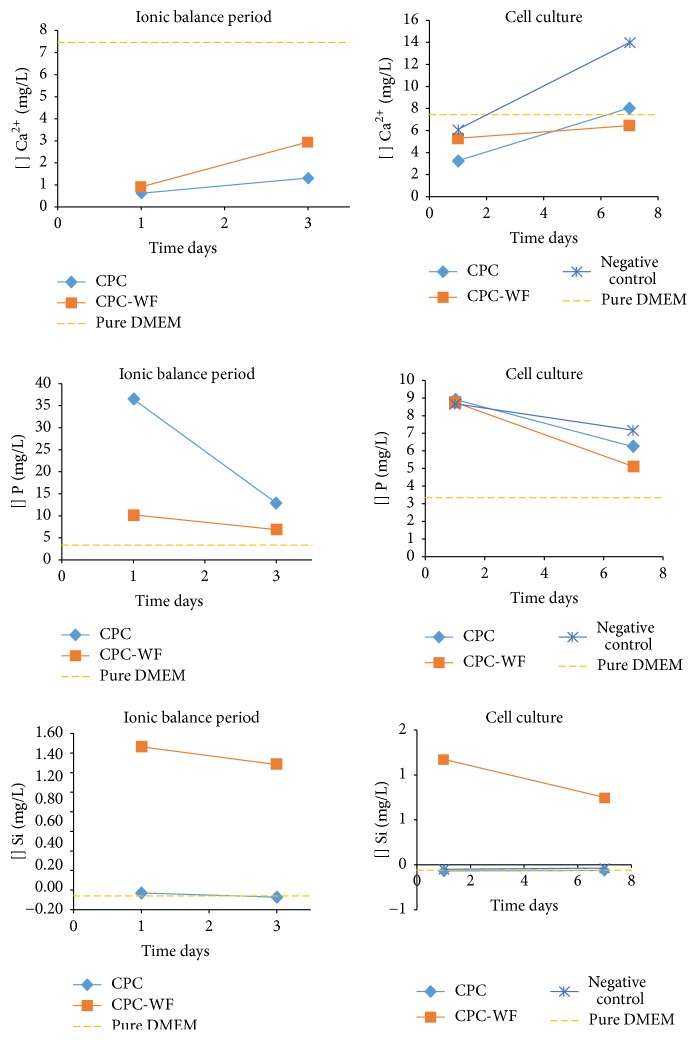
Ca^2+^, Si, and P ion concentrations in culture medium during the 3-day ionic balance period (quantifications at days 1 and 3) and under cell culture (days 1 and 7). DMEM alone was used as a control.

During the ion balance period, we noted a depletion of Ca^2+^ and release of P ions after 1 day of immersion in DMEM. On day 3 of the ion balance period, we detected an increase in Ca^2+^ concentration and reduction in the release of P ions. ICP-OES data indicated that the Ca^2+^ and P concentrations in CPC and CPC-WF samples were similar to those of the negative control after 3 days of ion balance in DMEM. There was a steady decrease in the rate of Si release from CPC-WF samples throughout the immersion period (Figure 4). All of the evaluated ions (Ca^2+^, Si, and P) were at similar concentrations after 7 and 14 days of culture. These data suggest that ion levels become balanced by day 7 of culture (Figure 4). For this reason, we omitted the day 14 data from Figure 4.

## 4. Discussion

*In vitro* analysis of biomaterials in cell culture is a valuable tool for understanding how recently developed materials elicit adverse reactions at the cellular level [15, 16]. Suitable biomaterials should be noncytotoxic and be able to maintain and stimulate cell differentiation [19]. Here, we evaluated the response of cultured osteoblast-like cells to WF-reinforced CPC.

We found that both CPC and CPC-WF enable cell adhesion, spreading, and increased viability over time. However, after 14 days in culture, cell viability was significantly higher on CPC-WF than CPC. Cell adhesion and proliferation depend on both the physical and chemical characteristics of their substrate [20]. CPC undergoes hydrolysis while in contact with aqueous solution, giving rise to a layer of apatite that resembles biological apatite. Such layer is a common characteristic of bioactive materials and enables binding to bone tissue, thus improving graft assimilation [21]. According to Chou et al. [16], this process is essential for the formation, growth, and maintenance of a tissue-biomaterial interface, as well as for proliferation and bone matrix synthesis. Morejón-Alonso et al. [22] demonstrated that addition of calcium silicate to a *α*-TCP-based cement improves bioactivity by hastening the formation of a dense apatite layer over the cement surface. Based on our data, together with the work of Morejón-Alonso et al. [22], we propose that hastened apatite layer formation could account for the increased cell viability of CPC-WF compared to CPC observed here.

In addition to the physical characteristics of CPC, which favor cell adhesion and proliferation, the chemical composition of cement also influences cellular responses. Mestres et al. [18] studied the ionic properties of CPC doped with silicon in culture medium and the influence of these ions on the response of osteoblast-like cells and observed calcium depletion in medium containing CPC. These authors also reported a delay in cell proliferation that was attributed to the material's strong ionic modification. Unlike Mestres et al. [18], we observed an increase in cell viability over time in both types of cement studied after the ionic balance period. In addition, as compared to CPC, the cell viability in CPC-WF was significantly increased. Other studies reported similar results about the role of silicon in osteoblast-like cell proliferation [11, 23]. Cell death after culture in calcium phosphates has been reported [24–26], which is generally attributed to changes in the ion concentration of the medium. According to Tamai et al. [27], the release of P from calcium phosphate-based materials induced the formation of phosphoric acids, leading to acidification of the culture medium. We also demonstrated that P release alters the pH of the medium, which changes from its usual red to a yellow appearance. Here, after the second medium change of the ion balance period, the culture medium pH remained stable (neutral). ICP-OES analyses found that, on day 3 of the ion balance period, there is a reduction in the release of P into the culture medium in the presence of CPC. Thus, we propose that the ion balance period used here facilitated an increase in cell viability during subsequent cell culture steps.

The ability to induce differentiation of osteoblast-like cells is fundamental to the success of biomaterials for bone tissue engineering. ALP is known to be an early marker of osteoblast differentiation [28]. This enzyme hydrolyzes phosphate substrates and results in the release of inorganic phosphate, which binds to calcium and forms hydroxyapatite [29]. Peak ALP activity generally corresponds to the onset of mineralization [30]. Our data demonstrate a direct link between the addition of WF in CPC and the production of ALP. The biological role of Si in bone metabolism is still unclear; several studies have shown that the presence of silicon in biomaterials stimulates bone formation [20, 31, 32] and enhanced ALP activity and expression [33, 34].

## 5. Conclusion

Taken together, our data suggest that CPC samples are likely to achieve ion balance with their culture medium over time. Only after this balance has been achieved, can cells be cultured and maintained for long periods. All types of CPC tested here were biocompatible for osteoblast-like cells. The addition of WF increased cell viability, as well as the activity of ALP, which could be directly related to silicon release in the medium. Our findings suggest that CPC-WF could be applied as a scaffold in bone tissue engineering strategies.

## Abbreviations

ALP: Alkaline phosphatase
CPC: Calcium phosphate cement
DMEM: Dulbecco's modified Eagle's medium
ICP-OES: Inductively coupled plasma optical emission spectroscopy
MTT: 3-(4,5-Dimethylthiazol-2-yl)-2,5-diphenyl tetrazolium bromide
PBS: Phosphate buffer saline
WF: * Wollastonite* fibers
*α*-TCP: *α*-Tricalcium phosphate.
